# Epidemiological Study of Dengue Fever in a Tertiary Care Hospital in Dera Ismail Khan, Pakistan

**DOI:** 10.7759/cureus.88950

**Published:** 2025-07-29

**Authors:** Ahtesham Khan, Muhammad Anan Khan, Kainat Fatima, Muhammad Fawad, Muhammad Hamza Khan, Ramla Rani, Taha Bukhari

**Affiliations:** 1 Department of General Medicine, District Headquarter Hospital, Medical Teaching Institute, Dera Ismail Khan, PAK; 2 Department of General Medicine, Mufti Mehmood Memorial Teaching Hospital, Medical Teaching Institute, Dera Ismail Khan, PAK; 3 Department of Anesthesiology and Critical Care, Combined Military Hospital, Abbottabad, PAK; 4 Department of General Medicine, Gomal Medical College, Medical Teaching Institute, Dera Ismail khan, PAK

**Keywords:** dengue fever, disease burden, epidemiology, mosquito control, pakistan, public health, seasonal variation, tertiary care hospital, vector-borne disease

## Abstract

Background

Dengue fever significantly burdens healthcare systems, particularly in resource-limited settings such as Dera Ismail Khan, Khyber Pakhtunkhwa, Pakistan. Mufti Mehmood Memorial Teaching Hospital, the designated Dengue Isolation Unit in the region, continues to receive a steady influx of patients. This study analyzed the epidemiological profile of dengue cases admitted to the hospital to support public health planning and guide resource allocation.

Methods

This retrospective study was conducted at Mufti Mehmood Memorial Teaching Hospital, Dera Ismail Khan, from October to December 2024, following ethical approval from the Institutional Review Board. A total of 168 patients with confirmed dengue fever (based on positive non-structural protein 1 (NS1) antigen or immunoglobulin M (IgM)/immunoglobulin G (IgG) antibody tests) were included. The hospital also serves neighboring districts, covering a combined population of over 4.2 million. Patients with co-infections (e.g., malaria, typhoid, or leptospirosis) or without consent were excluded. Data were collected using a structured abstraction form and included demographic variables (age, gender, district of residence), clinical features (fever, body aches, hemorrhagic signs, skin rash), epidemiological information (recent travel history and destination), and laboratory findings (NS1 antigen or IgM/IgG results). Statistical analysis was performed using IBM SPSS Statistics for Windows, Version 26.0 (Released 2018; IBM Corp., Armonk, NY, US). Associations between categorical variables were tested using the chi-square test, with p-values less than 0.05 considered statistically significant. Grammar was checked using Grammarly software (Grammarly, Inc., San Francisco, CA).

Results

Among the 168 patients diagnosed with dengue virus infection, 137 (81.5%) were male and 31 (18.5%) were female. The mean age was higher among females (34.5 ± 13.5 years) than males (28.0 ± 10.6 years), indicating more age variability in female patients. The most affected age group was 16-30 years (n = 101, 60.1%), followed by 31-45 (n = 44, 26.2%). Cases rose progressively from October (n = 37, 22%) to a peak in November (n = 108, 64.3%), before declining in December (n = 23, 13.7%). Serological testing showed full positivity for NS1, IgM, or IgG in all female patients. In contrast, a few male patients were negative for at least one marker (NS1, 4/137; IgM, 13/137; IgG, 9/137), though these differences were not statistically significant. Similarly, no significant gender-based differences were observed in clinical features such as fever, body aches, bleeding, or rashes. Most patients (n = 114, 67.9%) reported no recent travel history, while 54 (32.1%) had travelled to Karachi, Islamabad, or Rawalpindi. The highest number of cases originated from District Tank (n = 63, 37.5%), followed by Lakki Marwat (n = 46, 27.4%) and Dera Ismail Khan (n = 44, 26.2%).

Conclusion

Dengue fever peaks during the post-monsoon season and disproportionately affects males and young adults. District Tank and Lakki Marwat reported the highest burden of disease. Poor sanitation and limited mosquito control appear to drive transmission. These findings underscore the need for targeted vector control measures, public health awareness campaigns, and strengthened healthcare infrastructure to manage future outbreaks more effectively.

## Introduction

Dengue virus is a mosquito-borne viral infection primarily transmitted by *Aedes aegypti* and, to a lesser extent, *Aedes albopictus*. There are four dengue virus serotypes, meaning individuals can be infected up to four times. Dengue fever is suspected in patients presenting with signs and symptoms suggestive of the disease, such as a rapid onset of high-grade fever (≥38°C) lasting for 2-7 days, accompanied by headache, retro-orbital pain, muscle and joint pain, rash, and mucosal bleeding. Laboratory confirmation includes a positive non-structural protein 1 (NS1) antigen test or detection of immunoglobulin M (IgM) or immunoglobulin G (IgG) antibodies. Severe dengue remains a major cause of morbidity and mortality in parts of Latin America and Asia. Although no specific antiviral treatment exists, early diagnosis and supportive care can reduce mortality to below 1%. Dengue is endemic in tropical and subtropical regions, particularly in urban and semi-urban areas, where the global incidence has risen sharply, placing nearly half of the world’s population at risk. While an estimated 100-400 million infections occur annually, most cases are mild or asymptomatic [1,2].

Clinically, dengue ranges from asymptomatic infection to a flu-like illness and severe, life-threatening complications such as bleeding and shock [2]. The World Health Organization (WHO) has ranked dengue among the top 10 global health threats, reporting a 30-fold increase in incidence over the past five decades. WHO data show a rise in reported cases from 505,430 in 2000 to 5.2 million in 2019, with 6.5 million cases and more than 7,300 deaths reported by 2023 [3]. Before 1970, severe outbreaks were recorded in only nine countries, but dengue is now endemic in over 100 nations, including regions of Africa, the Eastern Mediterranean, Southeast Asia, the Western Pacific, and the Americas [4].

In the Middle East and North Africa, studies report a median seroprevalence of 25%, ranging from 0% to 62%, with the Red Sea region contributing the most data [5]. In Pakistan, dengue was first reported in 1982, with 12 confirmed cases out of 174 suspected. In Swabi, 62% of the reported cases were male, predominantly between 21 and 30 years of age [6]. Between 2006 and 2017, major outbreaks occurred in 2011 and 2013, primarily affecting the provinces of Punjab, Sindh, and Khyber Pakhtunkhwa [7]. A retrospective study conducted in Khyber Pakhtunkhwa reported a 20.6% positivity rate in 2017, with males (65.3%) more affected than females (34.7%). Peshawar recorded the highest burden, with infections peaking after the monsoon season, particularly in October (41%) and September (32%) [8]. Adults aged 21-40 years were the most affected group between June 2017 and May 2018. Of 2,000 suspected cases, 21% tested positive, with males accounting for 74% [9]. By September 2024, Pakistan had reported 2,795 new cases, with Balochistan being the most affected, followed by surges in Punjab, Sindh, and Khyber Pakhtunkhwa [10].

The rising global and regional burden of dengue fever underscores the need for epidemiological studies in resource-constrained areas like Dera Ismail Khan Division, where healthcare infrastructure is limited. Mufti Mehmood Memorial Teaching Hospital, the designated Dengue Isolation Unit for District Dera Ismail Khan, plays a central role in managing dengue cases and thus provides an appropriate setting for this research. The continued influx of patients places a considerable strain on the local healthcare system. This study aims to generate evidence-based data on the epidemiological characteristics of dengue patients admitted to the hospital, offering insights to inform public health strategies, optimize resource allocation, and strengthen the region’s capacity for effective dengue control.

## Materials and methods

Study design, setting, and duration

This retrospective study was conducted at Mufti Mehmood Memorial Teaching Hospital, Dera Ismail Khan, from October to December 2024. Medical records of patients admitted with confirmed dengue fever during this period were reviewed. Ethical approval was obtained from the Institutional Review Board of Gomal Medical College, Dera Ismail Khan (Reference No. 261/GJMS/JC), prior to data collection. A total of 168 laboratory-confirmed dengue cases were included, based on a positive NS1 antigen test or detection of IgM or IgG antibodies.

Study population, sample size, and eligibility criteria

Mufti Mehmood Memorial Teaching Hospital serves as the primary Dengue Isolation Unit for Dera Ismail Khan and also receives referrals from neighboring districts, including Tank, South Waziristan Tribal District (SWTD), and Lakki Marwat. The combined population of these districts is 4,229,635, based on the 2023 Census of Pakistan [1]. The required sample size (n = 168) was calculated using the Raosoft Sample Size Calculator, assuming a 95% confidence interval, a 6.17% margin of error, and a previously reported dengue prevalence of 21% [2,3]. The study included patients of all ages and genders with confirmed dengue fever. Patients were excluded if they declined consent or had co-existing infectious diseases such as malaria, typhoid, or leptospirosis, which could confound the diagnosis.

Data collection and analysis

Data were collected retrospectively using a structured data abstraction form and included the following variables: demographic information (age, gender, and district of residence), clinical features (fever, body aches, hemorrhagic manifestations, and skin rash), epidemiological factors (travel history within the past three weeks and travel destination), and laboratory findings (NS1 antigen test or detection of IgM or IgG antibodies). All patient data were anonymized to maintain confidentiality. Descriptive statistical analysis was conducted to assess the distribution of these variables and identify trends in the epidemiological and clinical profiles of dengue patients. Statistical analyses were performed using the IBM SPSS Statistics for Windows, Version 26.0 (Released 2018; IBM Corp., Armonk, NY, US). Frequencies and percentages were used for categorical variables, and associations between categorical variables were assessed using the chi-square test, with p-values less than 0.05 considered statistically significant. Additionally, Grammarly software (Grammarly, Inc., San Francisco, CA) was used to review the manuscript for grammar and spelling accuracy.

## Results

A total of 168 patients were confirmed to have dengue virus infection during the study period. Among these, 137 (81.5%) were male and 31 (18.5%) were female. The mean age of male patients was 28.0 ± 10.57 years, while female patients had a higher mean age of 34.5 ± 13.53 years, indicating that female patients were generally older and exhibited a wider age distribution, as reflected by the higher standard deviation. Patient ages ranged from below 15 years to over 60 years. The most affected age group was 16-30 years, accounting for 101 (60.1%) cases, followed by the 31-45 age group with 44 (26.2%) cases. Patients above 45 years accounted for 15 (8.9%) cases, while children aged 0-15 years comprised 8 (4.8%) cases. Monthly distribution showed a gradual increase in cases, with 37 (22%) reported in October, peaking in November with 108 (64.3%) cases, and declining to 23 (13.7%) cases in December. The distribution of gender, age groups, monthly case counts, and travel history is summarized in Table 1.

**Table 1:** Gender, age, monthly case distribution, and travel history of dengue patients

S. no.	Variables		Total (n = 168)	Percentage
1.	Gender	Male	137	81.5%
		Female	31	18.5%
2.	Age group	0-15 years	8	4.8%
		16-30 years	101	60.1%
		31-45 years	44	26.2%
		45-99 years	15	8.9%
3.	Month-wise distribution of dengue virus cases	October	37	22%
		November	108	64.3%
		December	23	13.7%
4.	Travel history	Yes	54	32.1%
		No	114	67.9%

Serological testing revealed that out of 168 patients, 164 (97.6%) were positive for NS1 antigen. This included 31 (18.5%) female patients and 133 (79.1%) male patients. The remaining 4 (2.3%) NS1-negative cases were all male. IgM antibodies were detected in 155 (92.2%) patients, comprising 31 (18.4%) females and 124 (73.8%) males, while 13 (7.7%) males tested negative. Similarly, IgG antibodies were positive in 159 (94.6%) patients, including all 31 (18.5%) females and 128 (76.1%) males, with 9 (5.3%) males testing negative. Notably, all female patients showed positivity for NS1, IgM, and IgG, whereas all negative serological results were observed exclusively among male patients. These findings indicate complete serological positivity among females, with some variability in serological markers observed in the male group, as detailed in Table 2.

**Table 2:** Serological profile of dengue patients by gender

Gender	NS1-positive, N (%)	NS1-negative, N (%)	IgM-positive, N (%)	IgM-negative, N (%)	IgG-positive, N (%)	IgG-negative, N (%)	Total, N (%)
Female	31 (18.5%)	0 (0%)	31 (18.5%)	0 (0%)	31 (18.5%)	0 (0%)	31 (18.5%)
Male	133 (79.1%)	4 (2.3%)	124 (73.8%)	13 (7.7%)	128 (76.1%)	9 (5.3%)	137 (81.5%)
Total	164 (97.6%)	4 (2.3%)	155 (92.2%)	13 (7.7%)	159 (94.6%)	9 (5.3%)	168 (100%)

The analysis of the association between gender and dengue serological markers (NS1, IgM, and IgG) yielded p-values of 0.336, 0.203, and 0.341, respectively. Since all values were above the 0.05 threshold for statistical significance, no significant association was found between gender and any of the dengue serological results. This indicates that the observed differences in positivity rates between male and female patients were not statistically meaningful. Although all female patients tested positive for NS1, IgM, and IgG, this finding is likely attributable to chance. Overall, gender did not appear to significantly influence dengue serological outcomes in this sample, as shown in Table 3.

**Table 3:** Association between gender and dengue serology Chi-square test of association was used to evaluate statistical relationships between categorical variables, with p-values < 0.05 considered statistically significant.

S. no.	Test	p-value
1	NS1 vs gender	0.336
2	IgM vs gender	0.203
3	IgG vs gender	0.341

Fever was a universal symptom, present in all 168 patients. Body aches were also commonly reported, affecting 136 (80.9%) males and all 31 (18.5%) females, with only 1 (0.6%) male not reporting this symptom. The p-value of 0.633 indicated no statistically significant difference between genders in the presence of body aches. Bleeding manifestations were observed in 24 (14.27%) males and 6 (3.5%) females, while they were absent in 113 (67.27%) males and 25 (14.96%) females. Again, the p-value of 0.236 showed no significant association with gender. Skin rashes were reported in 21 (12.47%) males and 1 (0.59%) female, but the p-value of 0.329 suggested no statistically significant gender difference. Overall, while slight variations were observed in the distribution of clinical features, such as bleeding and skin rash, between males and females, none of these differences were statistically significant. This suggests that the clinical presentation of dengue was largely similar across genders in this cohort, as shown in Table 4.

**Table 4:** Distribution of clinical features and their association with gender Chi-square test was used to assess the statistical significance of associations between categorical variables, with p-values < 0.05 considered statistically significant.

S. no.	Variables	Gender	Clinical features present, N (%)	Clinical features absent, N (%)	p-value
1.	Fever	Male	137 (81.5%)	0 (0%)	Fever is constant
		Female	31 (18.5%)	0 (0%)	
2.	Body aches	Male	136 (80.9%)	1 (0.6%)	0.633
		Female	31 (18.5%)	0%	
3.	Bleeding	Male	24 (14.27%)	113 (67.27%)	0.236
		Female	6 (3.5%)	25 (14.96%)	
4.	Skin rashes	Male	21 (12.47%)	116 (69.07%)	0.329
		Female	1 (0.59%)	30 (17.87%)	

**Figure 1:**
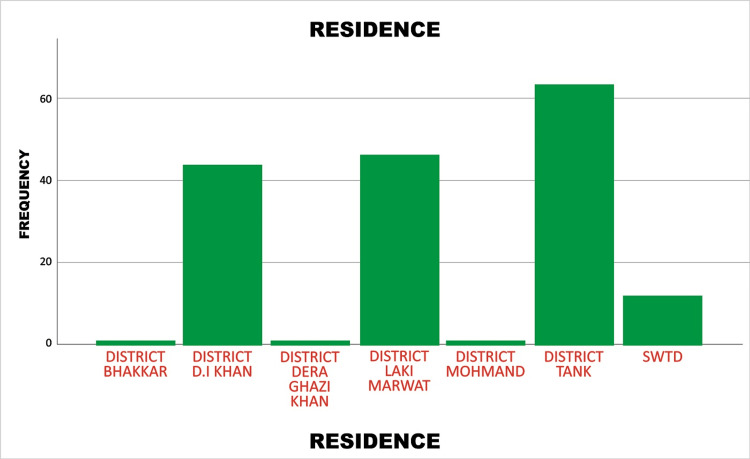
Frequency distribution of patient residences presenting to the Dengue Isolation Unit at Mufti Mehmood Memorial Teaching Hospital, Dera Ismail Khan, Khyber Pakhtunkhwa, Pakistan D.I: Dera Ismail, SWTD: South Waziristan Tribal District.

The highest number of dengue cases admitted to the Dengue Isolation Unit at Mufti Mehmood Memorial Teaching Hospital originated from District Tank, accounting for 63 (37.5%) out of 168 total cases. This was followed by District Lakki Marwat with 46 (27.4%) cases, and District Dera Ismail Khan with 44 (26.2%) cases. A smaller number of patients (12 (7.1%) cases) were admitted to the SWTD. Additionally, 1 (0.6%) case each was reported from District Bhakkar, Dera Ghazi Khan, and District Mohmand. These figures indicate that the majority of dengue patients came from District Tank, Lakki Marwat, and Dera Ismail Khan, as illustrated in Figure 1.

**Figure 2:**
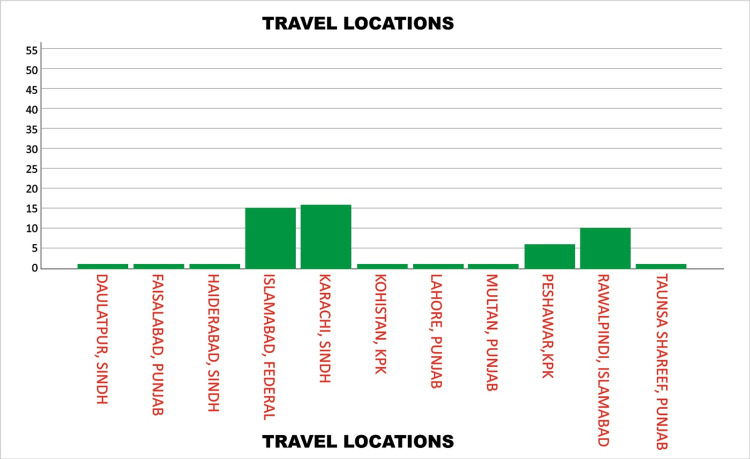
Distribution of travel destinations among dengue patients with recent travel history presenting to the Dengue Isolation Unit at Mufti Mehmood Memorial Teaching Hospital, Dera Ismail Khan, Khyber Pakhtunkhwa, Pakistan KPK: Khyber Pakhtunkhwa.

Regarding travel history, 54 (32.1%) patients reported travel within three weeks prior to symptom onset, consistent with the known dengue incubation period of 3-14 days. The remaining 114 (67.9%) had no recent travel history. Among those who had traveled, the most frequently reported destinations were Karachi with 16 (29.6%) cases, Islamabad with 15 (27.7%) cases, Rawalpindi with 10 (18.5%) cases, and Peshawar with 6 (11.1%) cases. Other cities such as Lahore, Kohistan, Multan, Faisalabad, Taunsa Sharif, Daulatpur, and Hyderabad were also reported but less frequently, each contributing one or two cases. These findings suggest that a significant proportion of dengue patients had recently traveled to major urban centers, particularly Karachi, Islamabad, and Rawalpindi, as shown in Figure 2.

## Discussion

Dengue is predominantly found in tropical and subtropical regions, affecting both urban and suburban areas. In Pakistan, dengue fever is a year-round, nationwide hazard, with most cases reported between September and December. The National Institute of Health, Islamabad, reported 22,938 cases of dengue fever in 2017, around 3,200 cases in 2018, 24,547 cases in 2019, 3,442 cases in 2020, and a total of 48,906 cases with 183 deaths countrywide in 2021 [4]. Over the last 15 years, specific regions of Pakistan have seen a tenfold increase in the reported cases of dengue [5].

The current study showed that the percentage of males was higher (137, 81.5%) compared to females (31, 18.5%). A similar study conducted in District Haripur in 2023 reported that male patients were 421 (58.8%), while female patients were 295 (41.2%) [6]. Another study conducted in the Khyber Pakhtunkhwa province of Pakistan in 2021 found that dengue fever was more common in males (65%) [7]. Similarly, according to a retrospective analysis from 2012 to 2019 in Punjab province of Pakistan, among the total 26,582 infected individuals, 18,381 (69.14%) were males, while 7,301 (27.46%) were females, which aligns with the findings of the current study [8]. The higher male infection rate may be attributed to greater male mobility, as most females in Pakistan tend to stay indoors, whereas males frequently travel and work outdoors, increasing their exposure to mosquito bites.

In the current study, the most affected age group was 16-30 years, accounting for 101 (60.1%) cases, followed by the 31-45 years age group with 44 (26.2%) cases. These findings are consistent with those from the 2023 dengue outbreak in Haripur, Pakistan, where the 16-30 years age group was also the most predominant, with 301 (42.0%) cases, followed by the 31-45 years age group with 184 (25.7%) cases [6]. In a similar study conducted in District Bannu, Khyber Pakhtunkhwa, Pakistan, in 2022, the most affected age group was 16-40 years, with 407 (56.84%) cases [9].

The higher occurrence of dengue fever in this age group is attributed to increased outdoor activities, particularly during the early morning and evening hours. The most common presenting symptoms were high-grade fever (168, 100%) and body aches (167, 99.4%). These findings are consistent with a study conducted during a dengue outbreak in Islamabad, Pakistan [10].

Peak incidences of dengue infection in the current study were observed in October with 37 (22%) cases, peaking in November with 108 (64.3%) cases, and declining in December with 23(13.7%) cases. The highest incidence occurred during the post-monsoon period. A similar trend was reported in the literature, where 94.2% (n = 113) of infections were noted after the rainy season [11]. This pattern has also been demonstrated by others, indicating that the spread of dengue fever is associated with monsoon rains [12]. Historical data shows a strong correlation between increased rainfall and rising dengue cases.

The primary reason for the surge in dengue incidence during the post-monsoon period is water stagnation, which creates ideal breeding conditions for mosquito vectors of the dengue virus [13]. In the present study, the highest number of cases was reported in District Tank with 63 (37.5%) cases, followed by District Lakki Marwat with 46 (27.4%) cases. Factors contributing to disease transmission in these areas include poor sanitation, contaminated drinking water, careless garbage disposal, overcrowding, and a lack of mosquito control strategies [13].

However, this study has certain limitations. It was conducted retrospectively at a single center, which may affect the wider relevance of the findings. Additionally, as the study relied on existing medical records, some variables may have been underreported or inconsistently documented. These factors should be taken into account when interpreting the results.

## Conclusions

Dengue fever remains a significant public health concern in Pakistan, with cases peaking after the monsoon season. This study found a higher prevalence among males (137; 81.5%) and individuals aged 16-30 years (101; 60.1%), likely due to increased outdoor exposure. The highest case burden was observed in District Tank and Lakki Marwat, areas affected by inadequate sanitation and poor mosquito control measures.

## Appendices

**Table 5:** Data of dengue patients PCR: polymerase chain reaction, PLT: platelet, MP: malarial parasite (peripheral smear).

Sr. no.	Gender	Age	High grade	Body aches	Bleeding	Skin rashes	Dengue PCR	PLT count	Dengue NS1	Dengue IgM	Dengue IgG	MP	Travel history
1	Male	25	Yes	Yes	No	No	No	317,000	Positive	Negative	Negative	Negative	Pezu
2	Male	18	Yes	Yes	No	Itching	No	92,000	Positive	Negative	Negative	Negative	Amakheil Tank
3	Male	21	Yes	Yes	No	Itching	No	108,000	Positive	Negative	Negative	Negative	Islamabad
4	Male	20	Yes	Yes	No	Itching	No	160,000	Positive	Negative	Negative	Negative	Karachi
5	Male	35	Yes	Yes	No	No	No	28,000	Positive	Positive	Negative	Negative	Swtd
6	Male	42	Yes	Yes	No	No	No	251,000	Positive	Negative	Positive	Negative	Pezu
7	Male	25	Yes	Yes	No	No	No	Not done	Positive	Negative	Negative	Negative	Pezu
8	Male	24	Yes	Yes	No	No	No	130,000	Positive	Negative	Negative	Negative	Dara Pezu 15 days back
9	Male	38	Yes	Yes	No	No	No	219,000	Positive	Negative	Negative	Negative	Peshawar
10	Male	18	Yes	Yes	Nose bleed	Yes	No	70,000	Positive	Negative	Negative	Negative	Tank
11	Male	35	Yes	Yes	No	No	No	252,000	Positive	Negative	Negative	Negative	Tank
12	Male	17	Yes	Yes	No	No	No	29,000	Positive	Negative	Negative	Negative	Haiderabad Sindh
13	Female	40	Yes	Yes	No	No	No	37,000	Positive	Negative	Negative	Negative	Tank
14	Female	13	Yes	Yes	No	No	No	336,000	Positive	Negative	Negative	Negative	Dara Pezu
15	Female	18	Yes	Yes	No	Itching	No	151,000	Positive	Negative	Negative	Negative	Dara Pezu
16	Female	27	Yes	Yes	No	No	No	102,000	Positive	Negative	Negative	Negative	Karachi
17	Male	20	Yes	Yes	No	No	No	141,000	Positive	Negative	Negative	Negative	Islamabad
18	Male	35	Yes	Yes	Yes	Yes	No	28,000	Positive	Negative	Negative	Negative	Rawalpindi
19	Male	35	Yes	Yes	No	No	No	44,000	Positive	Positive	Negative	Negative	Taunsa Shareef Punjab
20	Male	19	Yes	Yes	No	Severe itching	No	149,000	Positive	Negative	Negative	Negative	Rawalpindi
21	Male	55	Yes	Yes	No	No	No	366,000	Positive	Negative	Negative	Negative	Karachi
22	Male	21	Yes	Yes	No	No	No	144,000	Positive	Negative	Negative	Negative	Faisalabad
23	Male	23	Yes	Yes	No	No	No	68,000	Positive	Negative	Negative	Negative	Pezu
24	Male	25	Yes	Yes	No	No	No	120,000	Positive	Negative	Negative	Negative	Tank
25	Male	21	Yes	no	No	No	No	136,000	Positive	Negative	Negative	Negative	Rawalpindi
26	Female	40	Yes	Yes	No	No	No	107,000	Positive	Negative	Negative	Negative	Dara Pezu
27	Female	60	Yes	Yes	No	No	No	30,000	Positive	Negative	Negative	Negative	Dara Pezu
28	Female	40	Yes	Yes	No	No	No	75,000	Positive	Negative	Negative	Negative	Dara Pezu
29	Female	17	Yes	Yes	No	No	No	160,000	Positive	Negative	Negative	Negative	None
30	Female	45	Yes	Yes	No	No	No	157,000	Positive	Negative	Negative	Negative	Dara Pezu
31	Male	32	Yes	Yes	No	No	No	73,000	Positive	Positive	Negative	Negative	Islamabad
32	Male	28	Yes	Yes	No	No	No	69,000	Positive	Negative	Negative	Negative	None
33	Male	27	Yes	Yes	Yes (gums)	No	No	93,000	Positive	Negative	Negative	Negative	Dera Ismail Khan
34	Male	20	Yes	Yes	No	No	No	49,000	Positive	Negative	Negative	Negative	Rawalpindi
35	Male	30	Yes	Yes	No	yes	No	194,000	Positive	Negative	Negative	Negative	None
36	Male	18	Yes	Yes	No	yes	No	56,000	Positive	Negative	Negative	Negative	Rawalpindi
37	Male	25	Yes	Yes	No	No	No	37,000	Positive	Positive	Positive	Negative	None
38	Male	35	Yes	Yes	No	No	No	144,000	Positive	Negative	Negative	Negative	None
39	Male	25	Yes	Yes	Yes (gums)	yes	No	49,000	Positive	Negative	Negative	Negative	Islamabad
40	Male	28	Yes	Yes	No	No	No	76,000	Positive	Negative	Negative	Negative	Chakmalai (SWTD)
41	Male	26	Yes	Yes	No	No	No	84,000	Positive	Negative	Negative	Negative	Rawalpindi
42	Male	22	Yes	Yes	Yes (melena)	No	No	249,000	Positive	Negative	Negative	Negative	Kohistan
43	Male	15	Yes	Yes	No	No	No	163,000	Positive	Negative	Negative	Negative	None
44	Male	40	Yes	Yes	No	No	No	152,000	Positive	Negative	Negative	Negative	Islamabad
45	Male	16	Yes	Yes	No	No	No	315,000	Positive	Negative	Negative	Negative	None
46	Male	47	Yes	Yes	No	Known skin allergy	No	328,000	Positive	Negative	Negative	Negative	None
47	Male	32	Yes	Yes	No	No	No	117,000	Positive	Negative	Negative	Negative	Islamabad
48	Male	27	Yes	Yes	No	No	No	213,000	Positive	Negative	Negative	Negative	Islamabad
49	Male	17	Yes	Yes	No	No	No	21,000	Positive	Negative	Positive	Negative	None
50	Male	18	Yes	Yes	No	No	No	34,000	Positive	Negative	Negative	Negative	Islamabad
51	Male	19	Yes	Yes	No	No	No	18,000	Positive	Negative	Negative	Negative	Karachi
52	Male	20	Yes	Yes	No	yes	No	197,000	Positive	Negative	Negative	Negative	None
53	Male	27	Yes	Yes	Yes (in stool)	yes	no	19,000	Positive	Negative	Negative	Negative	Islamabad
54	Male	20	Yes	Yes	Yes (in stool)	No	No	259,000	Positive	Positive	Negative	Negative	Karachi
55	Male	22	Yes	Yes	No	No	No	46,000	Positive	Negative	Negative	Negative	Karachi
56	Male	16	Yes	Yes	Yes (in stool)	No	No	162,000	Positive	Negative	Negative	Negative	Rawalpindi
57	Male	25	Yes	Yes	No	No	No	102,000	Positive	Negative	Negative	Negative	Karachi
58	Male	60	Yes	Yes	No	No	No	101,000	Positive	Negative	Negative	Negative	Karachi
59	Male	22	Yes	Yes	Yes (in stool)	No	No	95,000	Positive	Negative	Negative	Negative	Peshawar
60	Male	20	Yes	Yes	No	No	No	180,000	Positive	Negative	Negative	Negative	None
61	Male	40	Yes	Yes	No	No	No	36,000	Positive	Negative	Negative	Negative	Peshawar
62	Male	37	Yes	Yes	No	No	No	74,000	Negative	Positive	Negative	Positive for vivax	None
63	Male	23	Yes	Yes	Yes (epistaxis)	No	No	190,000	Positive	Negative	Negative	Negative	Rawalpindi
64	Male	18	Yes	Yes	No	No	No	92,000	Positive	Negative	Negative	Negative	None
65	Male	25	Yes	Yes	No	No	No	317,000	Positive	Negative	Negative	Negative	Pezu
66	Female	34	Yes	Yes	Yes	No	No	155,000	Positive	Negative	Negative	Negative	None
67	Male	45	Yes	Yes	Yes	No	No	66,000	Positive	Negative	Negative	Negative	Islamabad
68	Male	40	Yes	Yes	Yes	No	No	196,000	Positive	Negative	Negative	Negative	None
69	Male	40	Yes	Yes	Yes	No	No	236,000	Positive	Negative	Negative	Negative	None
70	Male	30	Yes	Yes	Yes	No	No	122,000	Positive	Negative	Negative	Negative	None
71	Male	19	Yes	Yes	No	No	No	74,000	Positive	Negative	Negative	Negative	None
72	Male	31	Yes	Yes	No	No	No	215,000	Positive	Weakly positive	Weakly positive	Negative	None
73	Male	45	Yes	Yes	No	No	No	30,000	Positive	Negative	Negative	Negative	None
74	Male	21	Yes	Yes	No	No	No	233,000	Positive	Negative	Negative	Negative	Islamabad
75	Male	30	Yes	Yes	No	No	No	167,000	Positive	Negative	Negative	Negative	None
76	Male	30	Yes	Yes	No	No	No	107,000	Positive	Negative	Negative	Negative	None
77	Female	30	Yes	Yes	Yes	No	No	157,000	Positive	Negative	Negative	Negative	None
78	Female	26	Yes	Yes	Yes	No	No	207,000	Positive	Negative	Negative	Negative	None
79	Male	27	Yes	Yes	No	No	No	119,000	Positive	Negative	Negative	Negative	None
80	Male	16	Yes	Yes	No	No	No	273,000	Positive	Negative	Negative	Negative	None
81	Male	32	Yes	Yes	No	No	No	217,000	Positive	Negative	Negative	Negative	None
82	Female	50	Yes	Yes	yes	No	No	73,000	Positive	Negative	Negative	Negative	None
83	Male	32	Yes	Yes	No	No	No	151,000	Positive	Negative	Negative	Negative	Karachi
84	Male	20	Yes	Yes	No	No	No	97,000	Positive	Negative	Negative	Negative	None
85	Male	36	Yes	Yes	Yes (in stool)	No	No	37,000	Positive	Negative	Negative	Negative	None
86	Male	25	Yes	Yes	Yes (in stool)	No	No	55,000	Positive	Negative	Negative	Negative	Islamabad
87	Female	28	Yes	Yes	yes	No	No	207,000	Positive	Negative	Negative	Negative	None
88	Female	17	Yes	Yes	No	No	No	76,000	Positive	Negative	Negative	Negative	None
89	Male	23	Yes	Yes	Yes (gums)	yes	No	149,000	Positive	Negative	Negative	Negative	None
90	Male	27	Yes	Yes	No	No	No	20,000	Positive	Negative	Negative	Negative	Tank City
91	Male	53	Yes	Yes	No	No	No	89,000	Positive	Negative	Negative	Negative	Tajazai (Lakki)
92	Male	7	Yes	Yes	No	No	No	173,000	Positive	Negative	Negative	Negative	None
93	Male	13	Yes	Yes	No	No	No	230,000	Positive	Negative	Negative	Negative	None
94	Male	19	Yes	Yes	No	No	No	281,000	Positive	Negative	Negative	Negative	None
95	Male	22	Yes	Yes	No	No	No	119,000	Positive	Negative	Negative	Negative	Karachi
96	Male	33	Yes	Yes	No	No	No	71,000	Positive	Negative	Negative	Negative	None
97	Male	24	Yes	Yes	No	No	No	35,000	Positive	Negative	Negative	Negative	Peshawar
98	Male	20	Yes	Yes	No	No	No	205,000	Positive	Negative	Negative	Negative	None
99	Male	24	Yes	Yes	No	No	No	129,000	Positive	Negative	Negative	Negative	None
100	Male	24	Yes	Yes	Yes (epistaxis)	No	No	35,000	Positive	Negative	Negative	Negative	None
101	Male	23	Yes	Yes	No	No	No	235,000	Positive	Negative	Negative	Negative	No
102	Male	36	Yes	Yes	No	No	No	213,000	Positive	Negative	Negative	Negative	No
103	Male	27	Yes	Yes	No	No	No	27,000	Positive	Negative	Negative	Negative	No
104	Male	11	Yes	Yes	No	No	No	45,000	Positive	Positive	Negative	Negative	No
105	Male	24	Yes	Yes	No	No	No	221,000	Positive	Negative	Negative	Negative	Tehsil Tank
106	Male	28	Yes	Yes	No	No	No	133,000	Positive	Negative	Negative	Negative	No
107	Female	30	Yes	Yes	No	No	No	235,000	Positive	Negative	Negative	Negative	No
108	Female	30	Yes	Yes	No	No	No	208,000	Positive	Negative	Negative	Negative	No
109	Male	40	Yes	Yes	No	No	No	18,000	Positive	Positive	Positive	Negative	Islamabad
110	Female	31	Yes	Yes	No	No	No	45,000	Positive	Negative	Negative	Negative	No
111	Female	50	Yes	Yes	No	No	No	168,000	Positive	Negative	Negative	Negative	No
112	Male	42	Yes	Yes	No	No	No	105,000	Positive	Negative	Negative	Negative	Daulat Pur, Sindh
113	Male	40	Yes	Yes	No	No	No	75,000	Positive	Negative	Negative	Negative	No
114	Male	20	Yes	Yes	No	No	No	106,000	Positive	Negative	Negative	Negative	Karachi
115	Male	22	Yes	Yes	No	No	No	177,000	Positive	Negative	Negative	Negative	Islamabad
116	Male	60	Yes	Yes	No	No	No	66,000	Positive	Negative	Negative	Negative	No
117	Male	23	Yes	Yes	No	No	No	173,000	Positive	Negative	Negative	Negative	No
118	Female	35	Yes	Yes	No	No	No	238,000	Positive	Negative	Negative	Negative	No
119	Female	28	Yes	Yes	No	No	No	98,000	Positive	Negative	Negative	Negative	No
120	Male	11	Yes	Yes	No	No	No	313,000	Positive	Negative	Negative	Negative	No
121	Male	22	Yes	Yes	No	No	No	59,000	Positive	Negative	Negative	Negative	Islamabad
122	Male	33	Yes	Yes	No	No	No	37,000	Positive	Positive	Positive	Negative	No
123	Male	22	Yes	Yes	No	No	No	25,000	Positive	Negative	Negative	Negative	No
124	Male	25	Yes	Yes	No	No	No	166,000	Positive	Positive	Positive	Negative	Karachi
125	Male	22	Yes	Yes	Yes (epistaxis)	No	No	38,000	Positive	Negative	Negative	Negative	No
126	Male	50	Yes	Yes	No	No	No	14,000	Positive	Positive	Positive	Negative	No
127	Male	38	Yes	Yes	No	No	No	111,000	Positive	Negative	Negative	Negative	No
128	Female	60	Yes	Yes	No	No	No	37,000	Positive	Negative	Negative	Negative	No
129	Female	38	Yes	Yes	No	No	No	235,000	Positive	Negative	Negative	Negative	No
130	Male	21	Yes	Yes	No	Yes	No	232,000	Positive	Negative	Negative	Negative	Tehsil tank
131	Male	28	Yes	Yes	Yes (epistaxis)	Yes	No	193,000	Positive	Negative	Negative	Negative	No
132	Male	60	Yes	Yes	No	Yes	No	138,000	Positive	Negative	Negative	Negative	Dera Ismail Khan
133	Male	35	Yes	Yes	No	Yes	No	188,000	Positive	Negative	Negative	Negative	Dera Ismail Khan
134	Male	23	Yes	Yes	No	No	No	117,000	Positive	Negative	Negative	Negative	No
135	Male	21	Yes	Yes	No	No	No	100,000	Positive	Negative	Negative	Negative	No
136	Female	35	Yes	Yes	No	No	No	225,000	Positive	Negative	Negative	Negative	No
137	Female	50	Yes	Yes	No	No	No	250,000	Positive	Negative	Negative	Negative	No
138	Male	31	Yes	Yes	No	Yes	No	60,000	Positive	Negative	Negative	Negative	No
139	Male	27	Yes	Yes	No	No	No	208,000	Positive	Negative	Negative	Negative	No
140	Male	27	Yes	Yes	No	Yes	No	236,000	Positive	Negative	Negative	Negative	Dera Ismail Khan
141	Male	27	Yes	Yes	No	Yes	No	86,000	Positive	Negative	Negative	Negative	No
142	Male	42	Yes	Yes	No	No	No	150,000	Positive	Negative	Negative	Negative	Peshawar
143	Male	28	Yes	Yes	No	Yes	No	138,000	Positive	Negative	Negative	Negative	No
144	Female	25	Yes	Yes	No	No	No	263,000	Positive	Negative	Negative	Negative	Tank
145	Male	20	Yes	Yes	No	No	No	23,000	Negative	Negative	Negative	Negative	Lahore
146	Male	45	Yes	Yes	Yes	No	No	32,000	Positive	Negative	Negative	Negative	Tank
147	Male	24	Yes	Yes	No	No	No	42,000	Positive	Negative	Negative	Negative	Tank
148	Male	20	Yes	Yes	No	No	No	65,000	Positive	Negative	Negative	Negative	Rawalpindi
149	Male	22	Yes	Yes	No	No	No	96,000	Negative	Negative	Negative	Negative	Karachi
150	Female	10	Yes	Yes	No	No	No	231,000	Positive	Negative	Negative	Negative	Kawari tank
151	Male	24	Yes	Yes	No	No	No	112,000	Positive	Negative	Negative	Negative	Rawalpindi
152	Male	25	Yes	Yes	No	No	No	57,000	Positive	Negative	Negative	Negative	Multan
153	Male	30	Yes	Yes	No	No	No	78,000	Positive	Negative	Negative	Positive PV	Tank
154	Male	58	Yes	Yes	No	No	No	129,000	Positive	Negative	Negative	Positive PV	D.I. Khan
155	Male	25	Yes	Yes	No	No	No	14,000	Positive	Negative	Negative	Positive PV	Karachi
156	Female	50	Yes	Yes	No	No	No	180,000	Positive	Negative	Negative	Negative	Pezu
157	Female	45	Yes	Yes	Yes	No	No	234,000	Positive	Negative	Negative	Negative	Lachra
158	Male	45	Yes	Yes	Yes	No	No	36,000	Positive	Negative	Negative	Negative	Tank
159	Male	26	Yes	Yes	Yes	No	No	12,000	Positive	Negative	Negative	Negative	Tank
160	Female	16	Yes	Yes	No	No	No	46,000	Positive	Negative	Negative	Negative	Pezu
161	Female	50	Yes	Yes	No	No	No	146,000	Positive	Negative	Negative	Negative	Pezu
162	Male	27	Yes	Yes	No	No	No	114,000	Positive	Negative	Negative	Negative	Tank
163	Male	38	Yes	Yes	No	No	No	177,000	Positive	Negative	Negative	Negative	Tank
164	Male	19	Yes	Yes	No	No	No	94,000	Positive	Negative	Negative	Negative	Pezu
165	Male	45	Yes	Yes	Yes	No	No	45,000	Positive	Negative	Negative	Negative	Tank
166	Male	8	Yes	Yes	No	No	No	72,000	Positive	Negative	Positive	Negative	Karachi
167	Male	20	Yes	Yes	No	No	No	99,000	Positive	Negative	Negative	Negative	Karachi
168	Male	25	Yes	Yes	No	No	No	42,000	Positive	Negative	Negative	Negative	Peshawar
